# Synergistic Effect of Pyrvinium Pamoate and Azoles Against *Aspergillus fumigatus in vitro* and *in vivo*

**DOI:** 10.3389/fmicb.2020.579362

**Published:** 2020-11-03

**Authors:** Yi Sun, Lujuan Gao, Youwen Zhang, Ji Yang, Tongxiang Zeng

**Affiliations:** ^1^Department of Dermatology, Jingzhou Central Hospital, The Second Clinical Medical College, Yangtze University, Jingzhou, China; ^2^Department of Dermatology, Zhongshan Hospital Fudan University, Shanghai, China; ^3^Department of Dermatology, Zhongshan Hospital Fudan University, Xiamen, China; ^4^Department of Clinical Medicine, Yangtze University, Jingzhou, China

**Keywords:** pyrvinium, *Aspergillus fumigatus*, synergy, fungi, antifungal, resistance, azole

## Abstract

The effects of pyrvinium pamoate alone and in combination with azoles [itraconazole (ITC), posaconazole (POS), and voriconazole (VRC)] were evaluated against *Aspergillus fumigatus* both *in vitro* and *in vivo*. A total of 18 clinical strains of *A. fumigatus* were studied, including azole-resistant isolates harboring the combination of punctual mutation and a tandem repeat sequence in the Cyp51A gene (AFR1 with TR34/L98H and AFR2 with TR46/Y121F/T289A). The *in vitro* results revealed that pyrvinium individually exhibited minimal inhibitory concentration (MIC) of 2 μg/ml against AFR1 but was ineffective against other tested strains (MIC > 32 μg/ml). Nevertheless, the synergistic effects of pyrvinium with ITC, VRC, or POS were observed in 15 [83.3%, fractional inhibitory concentration index (FICI) 0.125–0.375], 11 (61.1%, FICI 0.258–0.281), and 16 (88.9%, FICI 0.039–0.281) strains, respectively, demonstrating the potential of pyrvinium in reversion of ITC and POS resistance of both AFR1 (FICI 0.275, 0.281) and AFR2 (FICI 0.125, 0.039). The effective MIC ranges in synergistic combinations were 0.25–8 μg/ml for pyrvinium, 0.125–4 μg/ml for ITC, and 0.125 μg/ml for both VRC and POS, demonstrating 4- to 32-fold reduction in MICs of azoles and up to 64-fold reduction in MICs of pyrvinium, respectively. There was no antagonism. The effect of pyrvinium–azole combinations *in vivo* was evaluated by survival assay and fungal burden determination in the *Galleria mellonella* model infected with AF293, AFR1, and AFR2. Pyrvinium alone significantly prolonged the survival of larvae infected with AF293 (*P* < 0.01) and AFR1 (*P* < 0.0001) and significantly decreased the tissue fungal burden of larvae infected with AFR1 (*P* < 0.0001). Pyrvinium combined with azoles significantly improved larvae survival (*P* < 0.0001) and decreased larvae tissue fungal burden in all three isolates (*P* < 0.0001). Notably, despite AFR2 infection was resistant to VRC or pyrvinium alone, pyrvinium combined with VRC significantly prolonged survival of both AFR1 and AFR2 infected larvae (*P* < 0.0001). In summary, the preliminary results indicated that the combination with pyrvinium and azoles had the potential to overcome azole resistance issues of *A. fumigatus* and could be a promising option for anti-*Aspergillus* treatment.

## Introduction

Invasive aspergillosis (IA) remains a frequent and lethal disease in high-risk immunocompromised individuals (Kontoyiannis and Bodey, 2002). The most frequent causative pathogen of IA is *Aspergillus fumigatus*. Antifungal drugs are limited to treatment options. Azoles are the mainstay of treatment and prevention of IA (Patterson et al., 2016). Nevertheless, azole resistance, especially in *A. fumigatus*, has increased alarmingly and is responsible for the high mortality rate of IA (van der Linden et al., 2011; Verweij et al., 2016; Perez-Cantero et al., 2020). Alternative therapeutic options include amphotericin B and echinocandins. However, limited efficacy or potential toxicity have restricted their use in IA. In addition, clinically significant amphotericin B resistance in *Aspergillus* spp. has been increasingly reported (Sterling and Merz, 1998; Arabatzis et al., 2011; Ozkaya-Parlakay et al., 2016). Therefore, drug repositioning in combination therapy might be a promising option.

Pyrvinium pamoate (PP), a quinoline-derived cyanine dye, was approved as an antihelmintic agent by Food and Drug Administration in 1955. PP is used to treat parasitic infections in humans (Beck et al., 1959; Wagner, 1963). Interestingly, PP has also been demonstrated to strongly inhibit the growth of fluconazole-resistant *Candida albicans* and potentiate the antifungal effect of fluconazole (Chen et al., 2015). In our previous study, PP was shown to exhibit antifungal activity alone [minimal inhibitory concentration (MIC) 2 μg/ml] and synergize with azoles against *Exophiala dermatitidis* both *in vitro* (Gao et al., 2018) and *in vivo* (Sun et al., 2020). Therefore, we speculate that PP might also exert some antifungal effect and positive interactions with conventional antifungals against *A. fumigatus*. Herein, the antifungal efficacy of PP alone and in combination with triazoles against *A. fumigatus* were investigated both *in vitro* and *in vivo*.

## Materials and Methods

### Fungal Strains, Antifungals, and Chemical Agents

Eighteen clinical *A. fumigatus* isolates were studied, including two isolates with the combination of punctual mutation and a tandem repeat sequence of the Cyp51A gene (TR46/Y121F/T289A and TR34/L98H). *Aspergillus flavus* (ATCC 204304) and *Candida parapsilosis* (ATCC 22019) were included for quality control. Morphologic and molecular identification of all isolates were performed via microscopy observation and sequencing of β-tubulin, calmodulin, and the internal transcribed spacer ribosomal DNA (Glass and Donaldson, 1995; Hong et al., 2005; Samson and Varga, 2009).

All four drugs, including PP, itraconazole (ITC), voriconazole (VRC), and posaconazole (POS), were purchased from Selleck Chemicals, Houston, TX, United States. Stock solutions were prepared by dissolving the drugs in dimethyl sulfoxide to achieve stock solutions of 3,200 μg/ml.

### *In vitro* Effect of Pyrvinium Pamoate Alone and Combined With Azoles Against *A. fumigatus*

The effects of PP alone and PP–azoles interactions against *A. fumigatus* were evaluated via the microdilution chequerboard technique, adapted from broth microdilution method M38-A2 (Clinical and Laboratory Standards Institute, 2008). Fungal cultures were grown on Sabouraud dextrose agar (SDA) for 7 days. Conidia were then harvested and suspended in sterile distilled water containing 0.03% Triton. The conidia suspension was diluted to a concentration of 1–5 × 10^6^ spores/ml and subsequently diluted in Roswell Park Memorial Institute 1640 medium to approximately 1–5 × 10^4^ spores/ml. Twofold serial dilutions of tested agents were prepared from stock solutions with Roswell Park Memorial Institute 1640 medium, according to M38-A2. The working concentration ranges were 0.06–32 μg/ml for PP and 0.03–16 μg/ml for azoles. As described, a 100-μl prepared conidia suspension was inoculated in each cell of the 96-well plate. Subsequently, the horizontal direction was inoculated with 50 μl of serial diluted PP, whereas the vertical direction was inoculated with another 50 μl of serially diluted azoles. The results were evaluated after incubation at 35°C for 48 h. The MICs were defined as the lowest concentration achieving complete inhibition of growth (Clinical and Laboratory Standards Institute, 2008). The interaction of drug combination was classified according to the fractional inhibitory concentration index (FICI). The FICI was calculated by the formula: FICI = (Ac/Aa) + (Bc/Ba), where Ac and Bc are the MICs of antifungals in combination, and Aa and Ba are the MICs of antifungals A and B alone, respectively, (Tobudic et al., 2010). A FICI of ≤0.5 indicates synergy, a FICI of >0.5 to ≤4 is classified as no interaction (indifference), whereas a FICI of >4 suggests antagonism (Odds, 2003). All tests were performed in triplicate.

### *In vivo* Efficacy of Pyrvinium Pamoate Alone and in Combination With Azoles in *Galleria mellonella*

The *in vivo* antifungal activity of PP alone and in combination with azoles against *A. fumigatus* infections was evaluated by *G. mellonella* survival assay as described previously (Maurer et al., 2015). Groups of 20 sixth instar larvae (∼300 mg, Sichuan, China) were maintained in the dark at room temperature before experiments. Fungal cultures of AF293, AFR1, and AFR2 were grown on SDA at 37°C for 72 h. Conidia were then harvested by gentle scraping of colony surfaces with sterile plastic loops, washed twice, and adjusted to 1 × 10^8^ spores/ml in sterile saline. For evaluation of the *in vivo* effects of PP alone and combined with azoles, the following intervention groups were included: PP group, ITC group, POS group, VRC group, PP with ITC group, PP with POS group, and PP with VRC group. Groups of larvae injected with 10-μl sterile saline or conidia suspension, and untouched larvae were served as control groups. Conidia suspension and therapeutic and control solutions were injected into the larvae via the last right proleg using a Hamilton syringe (25 gauge, 50 μl). Larvae were infected with fungal suspension 2 h before introducing therapeutic agents (0.5 μg per agent). All groups of larvae were incubated at 30°C in the dark. For survival studies, the death of larvae was monitored by visual inspection of the color (brown-dark/brown) every 24 h for a duration of 5 days. For tissue burden studies, three larvae from each group were selected without discrimination every 24 h for 4 days. Subsequently, selected larvae were suspended in 1 ml of saline-ampicillin and homogenized gently for a few seconds. The mix was 1,000-fold diluted with phosphate-buffered saline buffer, and 100 μl of the dilutions was inoculated on the SDA. The colonies were counted after incubation at 37°C for 24 h. The experiment was repeated triplicate using larvae from different batches.

### Statistical Analysis

Data were presented as mean ± SEM. Graph Pad Prism 7 was used for graphs and statistical analyses. The survival curves were analyzed by the Kaplan–Meier method. Tissue fungal burden was analyzed by analysis of variance. Differences were considered significant when *P* < 0.05.

## Results

### *In vitro* Effect of Pyrvinium Pamoate Alone and Combined With Azoles Against *A. fumigatus*

As shown in Table 1, the MIC ranges of PP alone were 2 μg/ml against AFR1 and >32 μg/ml against the other strains. The MIC ranges of azoles against azole-sensitive *A. fumigatus* were 1 μg/ml for ITC, 0.5 μg/ml for VRC, and 0.5–1 μg/ml for POS. The MIC ranges of azoles were >16 μg/ml for ITC, 4 μg/ml for VRC and POS against AFR1 (TR_34_/L98H), and >16 μg/ml for VRC, 4 μg/ml for ITC and POS against AFR2 (TR_46_/Y121F/T 289A).

**TABLE 1:** MICs and FICIs results with the combinations of PP and azoles.

**Strains**	**MIC^a^ (μg/ml) for**						
	
	**Agent alone**				**Combination^b^**		
	**PP**	**ITC**	**VRC**	**POS**	**PP/ITC**	**PP/VRC**	**PP/POS**
AF293	>32	1	0.5	1	0.5/1(1.008,I)	0.25/0.5(1.003,I)	1/0.125(0.141,S)
AF001	>32	1	0.5	0.5	2/0.25(0.281,S)	0.5/0.25(0.508,I)	0.5/0.25(0.508,I)
AF002	>32	1	0.5	1	2/0.25(0.281,S)	0.5/0.125(0.258,S)	0.25/0.125(0.129,S)
AF003	>32	1	0.5	1	0.25/1(1.003,I)	1/0.125(0.266,S)	1/0.125(0.141,S)
AF004	>32	1	0.5	1	8/0.25(0.375,S)	2/0.125(0.281,S)	0.5/0.125(0.132,S)
AF005	>32	1	0.5	1	8/0.125(0.25,S)	0.5/0.5(1.008,I)	0.5/0.125(0.132,S)
AF006	>32	1	0.5	1	2/0.125(0.156,S)	1/0.125(0.266,S)	0.5/0.125(0.132,S)
AF007	>32	1	0.5	1	0.5/1(1.008,I)	1/0.125(0.266,S)	0.5/0.125(0.132,S)
AF008	>32	1	0.5	1	4/0.125(0.188,S)	0.5/0.125(0.258,S)	1/0.125(0.141,S)
AF009	>32	1	0.5	1	4/0.25(0.313,S)	0.5/0.25(0.508,I)	0.25/0.125(0.129,S)
AF010	>32	1	0.5	1	1/0.25(0.266,S)	0.5/0.125(0.258,S)	0.5/0.125(0.132,S)
AF011	>32	1	0.5	1	2/0.25(0.281,S)	0.5/0.5(1.008,I)	0.5/0.125(0.132,S)
AF012	>32	1	0.5	1	1/0.25(0.266,S)	0.5/0.125(0.258,S)	1/0.125(0.141,S)
AF013	>32	1	0.5	1	2/0.25(0.281,S)	0.5/0.25(0.508,I)	0.25/0.125(0.129,S)
AF014	>32	1	0.5	1	4/0.25(0.313,S)	1/0.125(0.266,S)	0.5/0.5(0.508,I)
AF015	>32	1	0.5	1	4/0.25(0.313,S)	0.5/0.25(0.508,I)	0.5/0.125(0.132,S)
AFR1	2	>16	4	4	0.5/4(0.275,S)	0.5/2(0.75,I)	0.5/0.125(0.281,S)
AFR2	>32	4	>16	4	4/0.25(0.125,S)	16/16(0.75,I)	0.5/0.125(0.039,S)

*^*a*^MIC is the concentration resulting in 100% growth inhibition. ^*b*^FICI results are shown in parentheses. S, synergy (FICI of ≤0.5); I, no interaction (indifference; 0.5 < FICI ≤ 4). For FICI calculations, concentrations of 64 and 32 μg/ml were used when MICs were >32 and >16 μg/ml, respectively.*

When PP was combined with ITC, VRC, or POS, synergism was observed in 15 (83.3%, FICI 0.125–0.375), 11 (61.1%, FICI 0.258–0.281), and 16 (88.9%, FICI 0.039–0.281) strains of *A. fumigatus* (Table 1). Although AFR1 and AFR2 showed resistance to azoles, PP combined with ITC or POS showed favorable synergism against both strains. The effective MIC ranges of PP and ITC in synergistic combinations decreased to 1–8 and 0.125–0.25 μg/ml against azole-sensitive strains and 0.5–4 and 0.25–4 μg/ml against azole-resistant strains, respectively, (Table 1). In synergistic PP–POS combination, the MIC ranges of PP and POS against *A. fumigatus* decreased to 0.25–1 and 0.125 μg/ml, respectively. The effective working ranges of PP and VRC in synergistic combinations against *A. fumigatus* were 0.5–2 and 0.125 μg/ml, respectively, (Table 1). There was no antagonism observed in all combinations.

### *In vivo* Efficacy of Pyrvinium Pamoate Alone and in Combination With Azoles Against *A. fumigatus*

**FIGURE 1:**
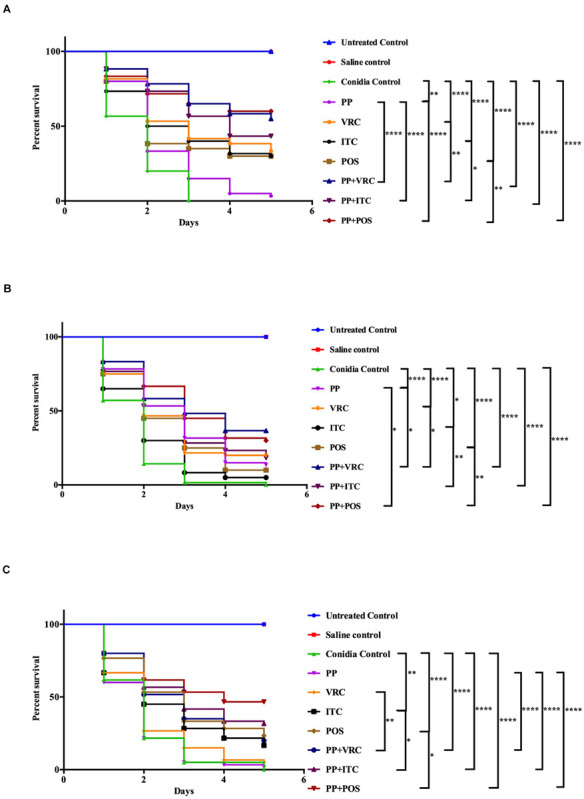
Survival curve of *A. fumigatus*-infected larvae with different interventions. **(A)** *G. mellonella* infected with AF293. Treatment with PP alone, azoles alone, or combined with PP all significantly improved larvae survival. Combination of PP with azoles acted synergistically against AF293 infection. **(B)** *G. mellonella* infected with AFR1. Treatment with PP alone, azoles alone, or combined with PP all significantly increased larvae survival. Combination of PP with azoles acted synergistically against AFR1 infection. **(C)** *G. mellonella* infected with AFR2. Larvae survival rates were significantly improved in groups treated with ITC alone, POS alone, and PP–azoles combinations. Larvae survival rates in the combination groups were significantly improved compared with azoles or PP alone groups (****P < 0.0001; **P < 0.01; and *P < 0.05).

For AF293-infected groups, the survival rates of larvae treated with PP, VRC, ITC, POS, PP with VRC, PP with ITC, and PP with POS were 3.3, 33.3, 30, 30, 55, 43, and 60%, respectively. Treatment with PP alone, azoles alone, and PP combined with azoles all significantly increased the survival rate of larvae (*P* < 0.01 for PP group, and *P* < 0.0001 for other groups; Figure 1A). The combination of PP with azoles acted synergistically against AF293 infection, compared with azoles alone or PP alone (*P* < 0.05).

Regarding AFR1 infection, the survival rates of larvae treated with PP, VRC, ITC, POS, PP with VRC, PP with ITC, and PP with POS were 13.3, 20, 5, 10, 36.7, 18.3, and 30%, respectively. PP alone, azoles alone, and azoles combined with PP all significantly improved the survival of larvae infected with AFR1 (*P* < 0.05 for ITC group, and *P* < 0.0001 for other groups; Figure 1B). The combination of PP with azoles acted synergistically against AFR1 infection, compared with the azoles alone group (*P* < 0.05). In addition, the survival of larvae in groups treated PP with VRC or POS was significantly higher than the PP alone group (*P* < 0.05).

With respect to AFR2 infection, the survival rates of larvae treated with PP, VRC, ITC, POS, PP with VRC, PP with ITC, and PP with POS were 1.7, 5, 16.7, 23.3, 20, 31.7, and 46.7%, respectively. AFR2 infections showed resistance to neither VRC nor PP treatment alone. Nevertheless, POS alone, ITC alone, and PP–azoles combinations all significantly increased the survival rate of AFR2-infected larvae (*P* < 0.01 for ITC group, and *P* < 0.0001 for other groups; Figure 1C). In addition, combination groups exhibited a significantly higher survival rate than azoles or PP treatment alone groups (*P* < 0.05).

### Fungal Burden Determination

**FIGURE 2:**
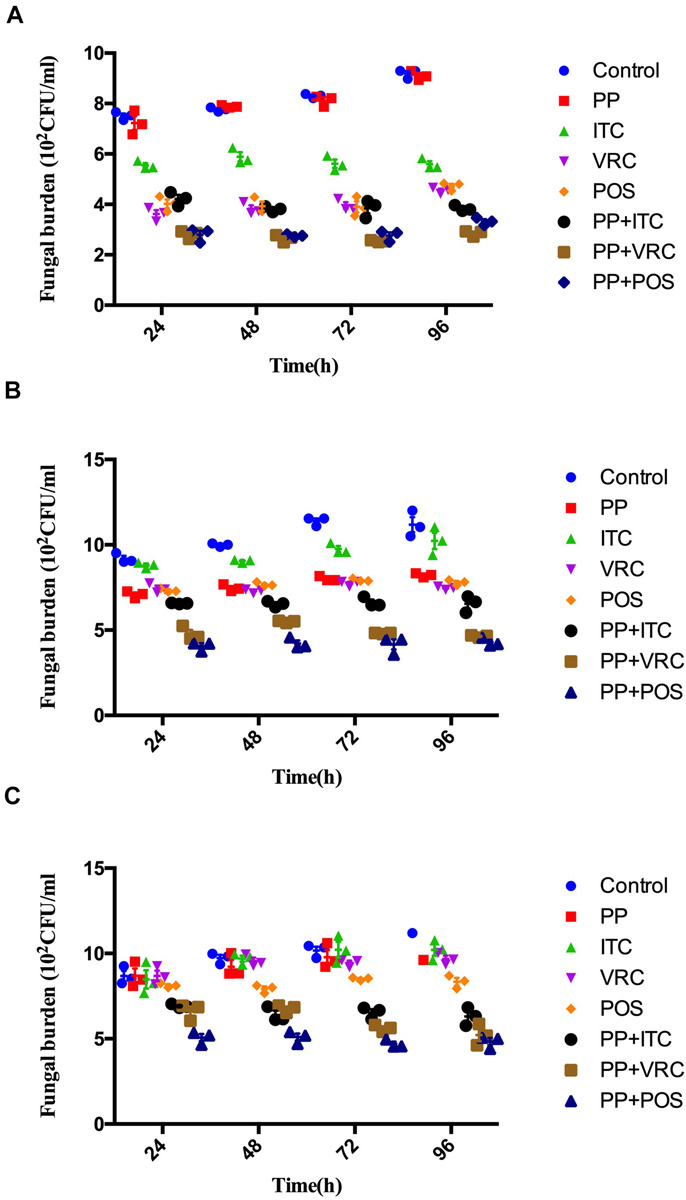
Effect of drug combinations on fungal burdens of larvae infected with *A. fumigatus.* **(A)** Larvae infected with AF293. All azole alone groups and combination groups exhibited significantly lower CFUs compared with control group (*P* < 0.0001). Groups treated with azoles in combination with PP exhibited significant (*P* < 0.0001) lower fungal burden than azole alone and PP alone group. **(B)** Larvae infected with AFR1. All groups exhibited significantly lower CFUs compared with control group (*P* < 0.01 for ITC group and <0.0001 for other groups). All combination groups exhibited significantly (*P* < 0.0001) lower fungal burden than azole alone and PP alone group. **(C)** Larvae infected with AFR2. POS group and all combination groups exhibited significantly lower CFUs compared with control group (*P* < 0.0001). All combination groups exhibited significant (*P* < 0.0001) lower fungal burden than azole alone and PP alone group.

Over the time of infection, increasing colony-forming unit (CFU) counting in larvae was observed (Figure 2). For larvae infected with AF293 (Figure 2A), the fungal burden of the PP alone group was comparable with the control group. The fungal burden in all azole groups was significantly lower than the PP group and control group (*P* < 0.0001). There was no significant difference between POS and VRC groups, whereas both groups exhibited significantly lower CFU counting than the ITC group (*P* < 0.001 for POS group versus ITC group, *P* < 0.0001 for VRC group versus ITC group). All combination groups exhibited significantly lower CFUs compared with the control group, azole alone group, and PP alone group (*P* < 0.0001).

As for larvae infected with AFR1 (Figure 2B), all intervention groups significantly decreased CFU number compared with the control group (*P* < 0.01 for the ITC group and *P* < 0.0001 for other groups). All combination groups exhibited significantly lower fungal burden than the azole alone and PP alone groups (*P* < 0.0001). The VRC group, POS group, and PP group exhibited comparable CFU counting, whereas the ITC group exhibited significantly higher CFU numbers compared with these three groups. Similarly, ITC combined with the PP group exhibited significantly higher fungal burden than PP combined with the POS or VRC group (*P* < 0.0001).

As for larvae infected with AFR2 (Figure 2C), the fungal burden of the PP group, ITC group, and VRC group were all comparable with the control group. There was no significant difference between the PP and azoles groups. However, the POS group significantly decreased CFU number compared with the ITC group (*P* < 0.001), VRC group (*P* < 0.05), and control group (*P* < 0.0001). All combination groups exhibited significantly lower CFUs compared with the control group, azole alone group, and PP alone group (*P* < 0.0001).

## Discussion

*Aspergillus* infections have been increasingly recognized as a global health challenge. Due to the occurrence of azole-resistant strains worldwide and limited antifungal options, the therapy of IA has been proved to be difficult (Chen et al., 2020). Combination therapies, which can expand the antifungal spectrum, improve therapeutic efficacy, and reduce adverse effects, could be a promising treatment option.

In the present study, PP was evaluated alone and combined with azoles against *A. fumigatus*. Although PP alone showed limited antifungal efficacy against most isolates tested *in vitro*, PP exhibited MIC of 2 μg/ml against azole-resistant strain AFR1. Accordingly, PP alone significantly improved the survival rate of larvae infected with AFR1 (*P* < 0.0001) and AF293 (*P* < 0.01), demonstrating the anti-*Aspergillus* effect of PP alone both *in vitro* and *in vivo*. Synergism between PP and ITC, VRC, or POS was observed in 15 (83.3%), 11 (61.1%), and 16 (88.9%) isolates *in vitro*. It was notable that PP potentiated the antifungal activity of both POS and ITC against AFR1 and AFR2. A fourfold and 16-fold reduction of ITC MICs against AFR1 and AFR2, respectively, and up to a 32-fold reduction of POS MICs against both AFR1 and AFR2 were observed, as shown in Table 1. Similarly, synergistic activity between PP and azoles resulted in up to an eightfold reduction of the MICs of azoles in azole-sensitive strains. The *in vitro* data were further confirmed *in vivo* because antifungal treatment with azoles and PP significantly improved larvae survival (*P* < 0.0001). Notably, despite AFR2 infection was resistant to VRC or PP alone and *in vitro* combination of PP-VRC showed inactive against both AFR1 and AFR2, PP combined with VRC significantly increased the survival of both AFR1 and AFR2 infected larvae (*P* < 0.0001). All azoles–PP combinations showed a significantly positive effect on larvae survival in comparison with the corresponding azole applied alone (*P* < 0.05). In accordance with *in vitro* susceptibility assay and *in vivo* survival assays, PP treatment alone significantly decreased CFU counting of larvae infected with AFR1. PP combined with azoles significantly decreased tissue fungal burden in larvae compared with PP alone and azole alone groups.

To date, mechanisms of azoles resistance in *A. fumigatus* are not fully characterized. The most investigated molecular mechanisms can be classified into the following two categories, namely *cyp51*-mediated resistance, including Cyp51 protein alterations and overexpression of the target enzyme, and non-*cyp51*-mediated resistance, including upregulation of efflux pump systems, fungal stress response, antifungal enzymatic degradation, biofilm formation, and alternative pathways activated to overcome the efficacy of antifungals (Wei et al., 2015; Hagiwara et al., 2016; Chen et al., 2020; Perez-Cantero et al., 2020). In these cases, azole resistance caused by a mutation in the Cyp51A gene combined with tandem repeats of the gene promoter region, e.g., TR_34_/L98H and TR_46_/Y121F/T289A, which leads to significant cyp51A overexpression, challenges the current understanding of the development of azole resistance in *A. fumigatus* (Mellado et al., 2007; van der Linden et al., 2013).

The TR_34_/L98H and TR_46_/Y121F/T289A were first reported in Netherlands in 1998 (Snelders et al., 2008) and 2009 (van der Linden et al., 2013), respectively. Up to date, isolates harboring these two mechanisms were found to be the major route for resistance cases and spread worldwide (Meis et al., 2016). It was estimated that 82–89% of azole-resistant cases in Netherlands were due to TR34/L98H and TR46/Y121F/T289A (Verweij et al., 2016), whereas this was the case in 64% of cases in Belgium (Vermeulen et al., 2015) and 87% of cases in Turkey (Ozmerdiven et al., 2015). Clinically, the role of azoles in aspergillosis caused by azole-resistant strains is very limited (Meis et al., 2016). Therefore, it is exciting to find that PP was able to reverse ITC and POS resistance of AFR1 and AFR2 *in vitro* and synergize with VRC, ITC, or POS *in vivo* against both AFR1 and AFR2. In addition, it is noteworthy that PP alone showed moderate antifungal effect against AFR1 both *in vitro* and *in vivo* but was inactive against AFR2 and other isolates tested. We suspected that non-*cyp51*-mediated factors might have played a critical role in the antifungal effect of PP against AFR1.

Previously, PP has been demonstrated to strongly inhibit the growth of the fluconazole-resistant i(5L) strain of *C. albicans*, which contains double copies of the left arm of chromosome 5 (Selmecki et al., 2006; Chen et al., 2015), and enhances the antifungal efficacy of fluconazole, demonstrating the efficacy of PP toward aneuploidy-based azole resistance. Acquisition of aneuploidy has been documented in pathogenic fungi *C. albicans*, *Saccharomyces cerevisiae*, and *Cryptococcus neoformans* (Todd et al., 2017). Specific environmental conditions, such as antifungal stress and fungal interactions with the host, might result in aneuploidy, which has been associated with drug resistance of fungi (Todd et al., 2017). Although the role of the aneuploid genome has not been described in *A. fumigatus* up to date, we suspected that ploidy changes might have occurred in *A. fumigatus*. In-depth studies are needed to investigate the probability of aneuploidy in *A. fumigatus*, especially AFR1 strain, and the possible role of aneuploidy in the anti-*Aspergillus* effect of PP. Furthermore, previous studies revealed that PP exhibited anticancer effects via mitochondrial respiration inhibition and CK1α activation, affecting multiple important biological processes and signaling pathways, such as Akt and Wnt-β-catenin-dependent pathways, autophagy, and energy (Momtazi-Borojeni et al., 2017). Therefore, we suspected that PP might also have some effect on critical fungal biological processes, such as stress response, biofilm formation, and drug efflux pump, as demonstrated in *E. dermatitidis* (Sun et al., 2020). However, further investigations are needed to elucidate the underlying mechanism.

In conclusion, the preliminary results indicated that PP could overcome azole resistance issues of *A. fumigatus* and might be a promising therapeutic strategy for anti-*Aspergillus* treatment. However, the limitation of the present study is the sample size of resistant strains of *A. fumigatus* studied. More isolates of resistant strains involving different phenotypes and genotypes are needed in the future study to help establish a comprehensive profile of the effect of PP alone and in combination with azoles against *A. fumigatus.*

## Data Availability Statement

The raw data supporting the conclusions of this article will be made available by the authors, without undue reservation.

## Ethics Statement

Written informed consent was obtained from the individual(s) for the publication of any potentially identifiable images or data included in this article.

## Author Contributions

LG and YS conceived and designed the study. YZ and YS performed all the experiments. LG analyzed the data and wrote the manuscript. JY and TZ provided general guidance and revised the manuscript. All authors contributed to the article and approved the submitted version.

## Conflict of Interest

The authors declare that the research was conducted in the absence of any commercial or financial relationships that could be construed as a potential conflict of interest.
